# Efficacy and safety of ciprofol for the induction of general anesthesia in patients with obesity undergoing laparoscopic sleeve gastrectomy: A double-blind randomized, controlled study

**DOI:** 10.1371/journal.pone.0329005

**Published:** 2025-07-24

**Authors:** Xiaowei Chi, Yi Xu, Qiang Li, Keshu Xia, Qiang Fu

**Affiliations:** 1 Department of Anesthesiology, The Third People’s Hospital of Chengdu, Affiliated Hospital of Southwest Jiaotong University, Chengdu, Sichuan, China; 2 Department of Anesthesiology, The First Affiliated Hospital of Chongqing Medical University, Chongqing, China; Massachusetts General Hospital, UNITED STATES OF AMERICA

## Abstract

**Background:**

The selection of safe and effective anesthetic agents for patients undergoing bariatric surgery is vital. This study aimed to evaluate the efficacy and safety of ciprofol in inducing general anesthesia in patients with obesity undergoing laparoscopic sleeve gastrectomy.

**Methods:**

A total of 212 patients scheduled for laparoscopic sleeve gastrectomy were randomly allocated into two groups in a 1:1 ratio: the ciprofol (0.5 mg/kg, n = 106) and propofol (2.5 mg/kg, n = 106) groups. The primary endpoint was to assess the success rate of anesthesia induction. Secondary endpoints included evaluating the time of induction, loss of eyelash reflex, changes in bispectral index, and adverse event incidence.

**Results:**

The success rates of anesthesia induction were 100% in both groups. Ciprofol demonstrated non-inferiority to propofol in induction success. The times to successful induction onset and eyelash reflex disappearance were significantly longer in the ciprofol group compared to those in the propofol group (39.38 ± 8.57 s vs. 36.74 ± 6.82 s, P = 0.014 and 40.36 ± 8.59 s vs. 37.77 ± 6.84 s, P = 0.016, respectively). The adverse events incidence was significantly lower in the ciprofol group compared to that in the propofol group (25.47% vs. 89.62%, P = 0.000). The number of patients requiring top-up doses was not statistically significant (3.77% vs. 7.55%, P = 0.235). Ciprofol demonstrated advantages in hemodynamic stability and maintaining a better sedation level post-induction. Ciprofol was associated with a significantly lower incidence of hypotension compared to propofol (14.15% vs. 37.74%, P < 0.001), and more patients maintained appropriate sedation depth (86.80% vs. 72.64%, P = 0.010, 40 ≤ bispectral index ≤ 60 within 10 min of intravenous administration).

**Conclusion:**

Ciprofol offers a better sedative effect, fewer adverse events, and greater hemodynamic stability during general anesthesia induction in patients with obesity undergoing laparoscopic sleeve gastrectomy.

**Trial registration:**

ClinicalTrials.gov NCT05522998

## Introduction

Recently, obesity prevalence has increased globally [1], which has positioned bariatric surgery, including laparoscopic sleeve gastrectomy (LSG), as a key treatment strategy [2]. Morbid obesity leads to pathophysiological changes affecting metabolic, cardiovascular, and respiratory systems, thus increasing anesthetic risk [1]. This results in prevalent perioperative complications, such as circulatory depression, atelectasis, and hypoxemia [3]. Therefore, selecting safe and effective anesthetic agents for bariatric surgery patients is vital.

Propofol is widely used for anesthesia induction and maintenance, known for its rapid onset and recovery with minimal residual effects [4]. However, it can cause dose-dependent circulatory and respiratory depression, as well as injection pain, thereby increasing the incidence of adverse clinical drug reactions [5]. A rare but severe complication, propofol infusion syndrome, may induce metabolic disturbances, multiple organ failure, and, in extreme cases, death [6].

Ciprofol, an innovative intravenous anesthetic developed in China, is a potent, short-acting agonist of the γ-aminobutyric acid (GABA) receptor with dual anesthetic and sedative effects. Compared to propofol, it demonstrates greater potency [7]. Preliminary studies demonstrated ciprofol’s advantageous profile, including high potency, rapid onset and recovery, absence of accumulation, and low respiratory and circulatory depression post-injection, indicating considerable clinical potential [8,9].

However, there is limited experience with the clinical application of ciprofol in patients with obesity. The objective of this study was to compare the anesthetic effects and adverse reactions between ciprofol and propofol, as well as evaluate the effectiveness and safety of ciprofol for inducing general anesthesia in patients with obesity undergoing LSG.

## Materials and methods

### Research ethics and study design

The present randomized, double-blind, controlled study was conducted at the Third People’s Hospital of Chengdu, affiliated to Southwest Jiaotong University. The hospital’s Medical Ethics Committee approved the study (review board number: 2021S-109), and the trial was registered in ClinicalTrials.gov (Registration number: NCT05522998) on August 29, 2022. Written informed consent was obtained from all participants in accordance with the relevant ethical guidelines.

### Patients

The study’s participant selection adhered to rigorous inclusion and exclusion criteria. Eligible individuals were aged 19 − 65 years, with a body mass index (BMI) ≥35 kg/m^2^. They had an American Society of Anesthesiologists (ASA) physical status ranging from I to III and had been scheduled for LSG under general anesthesia at the Third People’s Hospital of Chengdu. Comprehensive criteria for both inclusion and exclusion can be found in S1 File.

### Sample size and power

We compared the efficacy of ciprofol (experimental group) with propofol (control group) in terms of the success rate for inducing general anesthesia, using a Type I error (false positive) rate of 0.025 (one-sided) and a test power of 80%. Based on a preliminary, unpublished study involving 25 patients with obesity, the success rate for inducing general anesthesia using study medications was 96%, and the non-inferiority margin was set at 8% based on available literature [10,11]. Using the methodology of Chow et al. [12] and calculations in the R language, the sample size was determined to be 95 participants per group. Considering a potential 10% loss due to follow-up issues or refusal to participate, the required sample size was increased to at least 106 individuals per group, resulting in a total sample size of 212 cases.

### Randomization and blinding

The simple randomization method was employed. The statistical team members generated 212 random numbers using SPSS 25.0 software (IBM, Chicago, IL, USA) and randomly assigned them into two groups: ciprofol (experimental group) and propofol (control group). A biostatistician, blinded to patient details, prepared sealed opaque envelopes containing each patient’s random number and group. On the day of the surgery, these envelopes were opened by two researchers not involved in data collection or analysis, who then prepared the study medications based on the enclosed information. Anesthesiologists, unaware of the groups, administered the anesthesia. All data were collected by an independent researcher who was not involved in syringe preparation or data analysis. This ensured the blinding of patients, anesthesiologists, outcome investigators, and the statistician to group allocation, thus maintaining the study’s blinding integrity.

### Blinding protocol

Drug Preparation: All study medications (ciprofol 0.5 mg/kg and propofol 2.5 mg/kg) were prepared by two independent researchers not involved in data collection or analysis. Both medications were diluted to identical volumes using 0.9% saline, thus ensuring indistinguishable physical appearance (milky-white emulsion).

Syringe Labeling: Prepared syringes were labeled with a unique randomization code (“Group A” or “Group B”) rather than drug names.

Administration Protocol: The anesthesiologist responsible for drug administration was instructed to avoid discussing the syringe contents or perceived drug effects with other team members. To maintain blinding, the anesthesiologist administering the induction agents was prohibited from participating in assessments or data collection.

### Study procedures

(1) Researchers prepared study medications and other anesthesia-inducing medications based on each patient’s lean body weight (LBW) and ideal body weight (IBW). The dosages of propofol/ciprofol, midazolam, and sufentanil were calculated using LBW for anesthesia induction [13]. Rocuronium dosage was based on IBW [14]. The following formulas were applied: (1 inch = 2.54 cm) [15,16].

BMI(kg/m2)=weight(kg)÷height2(m2)

LBW(kg)for\ males=9720×TBW/(6680+216×BMI),where\ TBW\ is\ total\ body\ weight

LBW(kg)for\ females=9720×TBW/(8780+244×BMI)

IBW(kg)for\ males=50+2.3×([length\ in\ inches–60])

IBW(kg)for\ females=45.5+2.3×([length\ in\ inches–60])

(2) Upon entering the operating room, a nurse set up a peripheral intravenous infusion line for the patient. Meanwhile, vital signs, including electrocardiogram (ECG), heart rate (HR), pulse oxygen saturation (SpO_2_), systolic blood pressure (SBP), diastolic blood pressure (DBP), mean arterial pressure (MAP), and bispectral index (BIS), were continuously monitored. The assessment of sedation levels was conducted using the Modified Observer’s Assessment of Alertness/Sedation (MOAA/S) scale, as presented in S2 File. Thereafter, patients underwent preoxygenation, breathing spontaneously with a mask delivering 100% oxygen at a flow rate of 4 L/min.
(3) Anesthesia induction: Two minutes before administering the study medications, patients were administered intravenous midazolam (0.04 mg/kg) within 15 seconds and sufentanil (0.4 µg/kg) within 30 seconds. Subsequently, they were administered an intravenous injection of either ciprofol (0.5 mg/kg) or propofol (2.5 mg/kg) within 30 seconds. If a MOAA/S score of ≤1 was not achieved within 1 minute post-initial dose, a supplementary half-dose of the initial propofol/ciprofol dose was administered over 10 seconds. A secondary supplementary dose was provided as rescue medication if a MOAA/S score of ≤1 was not achieved within 2 minutes. Failure to reach MOAA/S score of ≤1 within 3 minutes deemed the study medication unsuccessful for inducing general anesthesia, leading the anesthesiologist to induce anesthesia based on clinical judgment. Once achieving a MOAA/S score of ≤1, as indicated by the absence of response to mild stimulation or shaking, rocuronium (0.6 mg/kg) was administered intravenously within 15 seconds, followed by tracheal intubation after confirming skeletal muscle relaxation.

Baseline MOAA/S measurements were obtained prior to the administration of midazolam and re-assessed every 30 seconds thereafter. Injection pain was evaluated at 5-second intervals post-study medication administration. The time to eyelash reflex disappearance, starting from the initiation of the study medication was measured every 5 seconds using a sterile cotton swab. During anesthesia induction, vital parameters, including ECG, HR, SpO_2_, SBP, DBP, MAP, and BIS, were closely monitored and recorded while closely observing the patients for any adverse events.

### Outcome measurements

Trial data were collected by researchers who were blinded to the group allocation. These data included baseline characteristics, such as age, sex, height, TBW, LBW, BMI, ASA classification, prior anesthesia history, alcohol consumption habits, and comorbidities.

### Primary outcome

The primary endpoint was the success rate of general anesthesia induction, characterized by the proportion of successful cases in each group. The criteria for successful induction of anesthesia were: (1) MOAA/S score of ≤1 after administering study medication (none or no more than two additional doses of study medication) and (2) no use of alternative anesthetic agents.

### Secondary outcomes

The secondary outcomes of the study were: (1) time to successful induction from initial administration of the study medication, (2) time to disappearance of eyelash reflex from initial administration of the study medication, (3) BIS changes following anesthesia initiation, (4) hemodynamic changes after anesthesia initiation, and (5) use of supplementary doses of the study medication.

### Safety indicators adverse events

Safety indicators included: (1) injection-site pain (patients were enquired about arm pain during drug injection before they lost consciousness), (2) allergic reaction, (3) bradycardia (HR ≤ 55 beats/min, lasting >30 seconds), (4) tachycardia (HR ≥ 100 beats/min, lasting >30 seconds), (5) hypotension (SBP < 90 mmHg or a decrease of at least 30% from baseline), (6) hypertension (SBP ≥ 160 mmHg or an increase of at least 20% from baseline), (7) hypoxemia (oxygen saturation <90%, lasting >30 seconds), and (8) intubation response (unconscious swallowing, coughing, body movement, or tears).

### Adverse events that require special attention

These included (1) incidence of blood pressure reduction requiring treatment during anesthesia, (2) incidence of respiratory depression, (3) incidence of deep or shallow anesthesia, and (4) incidence of intraoperative awareness.

### Statistical analysis

Primary efficacy was analyzed using the Newcombe-Wilson scoring method [17]. Differences in the success rate of inducing general anesthesia and bilateral-sided 95% confidence intervals (CI) were evaluated. If the lower limit of the 95% CI for the success rate was greater than −8%, ciprofol at a dose of 0.5 mg/kg was deemed non-inferior to propofol at a dose of 2.5 mg/kg.

The statistical analysis of other efficacy endpoints was conducted using SPSS 25.0 software. Continuous numeric variables are presented as mean ± standard deviation, and inter-group comparisons were performed using the two independent samples t-test. Categorical variables are expressed as numbers and percentages (n [%]), and the chi-squared test or Fisher’s exact test was used for comparing groups. The differences were deemed statistically significant with a two-sided p-value <0.05. Repeated measures data were analyzed using a two-way repeated-measures ANOVA framework, with treatment group (ciprofol vs. propofol) and timepoint as between- and within-subjects factors, respectively. Sphericity was assessed via Mauchly’s test, with Greenhouse-Geisser corrections applied when violations were detected (ε < 0.75, P < 0.05).

## Results

**Fig 1:**
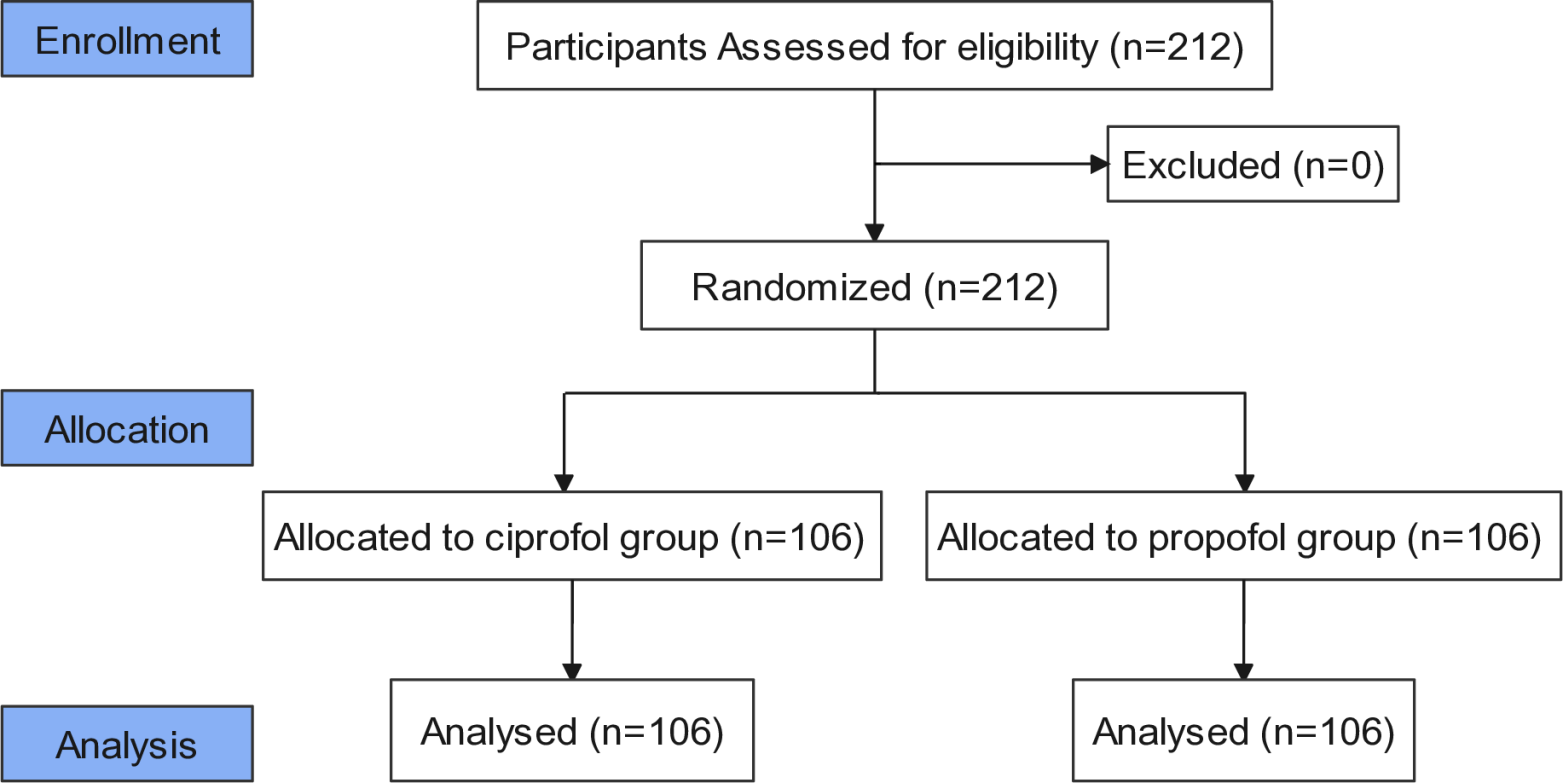
CONSORT flow diagram.

A total of 212 participants (106 in each group) were included in this study conducted between January and June 2023. All enrolled patients successfully completed the trial, and statistical analysis was performed using data from all participants (Fig 1). Baseline and demographic characteristics were similar across both groups, showing no significant differences (Table 1).

**Table 1:** Baseline and demographic characteristics of the study participants.

Baseline characteristics		Ciprofol group (n = 106)	Propofol group (n = 106)
Age, years	Mean ± SD	31.45 ± 7.85	32.65 ± 7.33
Sex, n (%)	Male	37 (34.91%)	30 (28.30%)
	Female	69 (65.09%)	76 (71.70%)
Height (cm)	Mean ± SD	166.30 ± 9.78	164.72 ± 8.42
Weight (kg), TBW	Mean ± SD	118.23 ± 20.93	113.84 ± 18.44
BMI (kg/cm^2^)	Mean ± SD	42.58 ± 5.32	41.82 ± 4.37
Weight (kg), LBW	Mean ± SD	61.71 ± 13.16	58.99 ± 11.61
ASA classification, n (%)	Class II	41 (38.68%)	46 (43.40%)
	Class III	65 (61.32%)	60 (56.60%)
Anesthesia history, n (%)	Yes	58 (54.72%)	54 (50.94%)
	No	48 (45.28%)	52 (49.06%)
Alcohol drinking, C	Yes	44 (44.51%)	40 (37.74%)
	No	62 (58.49%)	66 (62.26%)
Comorbidities			
HBP history, n (%)		27 (25.47%)	25 (23.58%)
DM history, n (%)		17 (16.04%)	23 (21.70%)
Midazolam-induced dose, mg	Mean ± SD	2.48 ± 0.52	2.36 ± 0.45
Sufentanil-induced dose, ug	Mean ± SD	27.9 ± 5.08	26.79 ± 4.06

BMI, body mass index; ASA, American Society of Anesthesiologists; HBP, high blood pressure; DM, diabetes mellitus; SD, standard deviation.

### Primary outcome

The success rate of general anesthesia induction was 100% in both groups. The difference in the success rates between the groups was 0%, with a 95% CI ranging from −3.50% to 3.50% (Table 2).

**Table 2:** Comparison of anesthesia induction success rates.

	Success rate induction^a^, n (%)	Difference (%)	95% CI
Ciprofol group	106 (100%)	0	(−3.50%, 3.50%)
Propofol group	106 (100%)	0	

^a^The success rate of anesthesia induction was analyzed using the Newcombe-Wilson scoring method. Differences in the success rates of anesthesia induction and bilateral-sided 95% CI were evaluated. The lower limit of the 95% CI for the difference in anesthesia induction success rates was > −8%. Data are expressed as n (%). CI, confidence interval.

### Secondary outcomes

The time to successful induction onset was significantly longer in the ciprofol group compared to the propofol group (39.38 ± 8.57 s vs. 36.74 ± 6.82 s, P = 0.014), as was the time to eyelash reflex disappearance (40.36 ± 8.59 s vs. 37.76 ± 6.84 s, P = 0.016) (Table 3). The average number of study medication administrations was 1.04 and 1.08 in the ciprofol and propofol groups, respectively, with no significant difference observed (P = 0.214). The majority of patients did not require additional doses. Only a small percentage of patients received additional doses: four (3.77%) and eight (7.55%) in the ciprofol and propofol groups, respectively, with no statistically significant difference observed (P = 0.235) (Table 3).

**Table 3:** Outcomes.

	Ciprofol group (n = 106)	Propofol group (n = 106)	P-value
Time to onset of successful induction (s), (Mean ± SD)	39.38 ± 8.57	36.74 ± 6.82	0.014^*^
Time to the disappearance of eyelash reflex (s), (Mean ± SD)	40.36 ± 8.59	37.76 ± 6.84	0.016^*^
Number of administrations, (Mean ± SD)	1.04 ± 0.19	1.08 ± 0.27	0.214
Number of patients given top-up doses			
First administration only (n, %)	102 (96.23%)	98 (92.45%)	0.235
First additional administration only (n, %)	4 (3.77%)	8 (7.55%)	0.235
Second additional administration (n, %)	0	0	
40 ≤ BIS ≤ 60 within 10 min of intravenous administration (n, %)	92 (86.80%)	77 (72.64%)	0.010^*^

* P < 0.05 vs. Propofol group.

SD, standard deviation; BIS, bispectral index.

**Fig 2:**
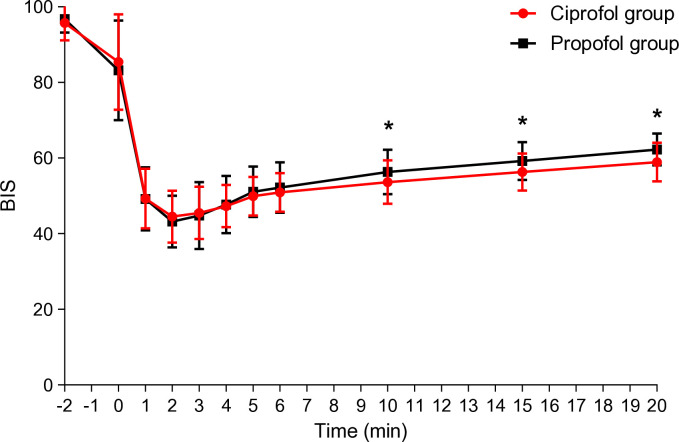
Changes in the bispectral index (BIS) following anesthesia induction; * P < 0.05 vs. Propofol group.

The changes in BIS during anesthesia induction for both groups are depicted in Fig 2. Within the first 20 minutes of general anesthesia induction, the objective sedation level, as assessed by BIS value changes, was evaluated. In the initial 8 minutes, the sedation level changes observed in the ciprofol group exhibited a similar pattern to those observed in the propofol group. However, from 8 to 20 minutes, the ciprofol group showed significantly lower BIS values (P < 0.05) (Fig 2). Additionally, a higher proportion of patients in the ciprofol group consistently maintained BIS scores below 60 throughout this period (P < 0.05) (Table 3).

### Safety indicators and adverse events

**Fig 3:**
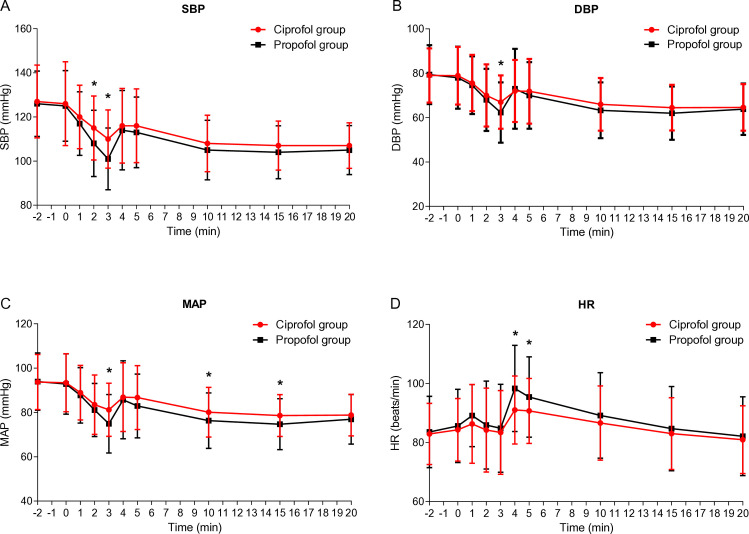
Changes in blood pressure and heart rate following anesthesia induction. Time 0 was defined as the baseline value 10 s prior to the administration of the study medication; * P < 0.05 vs. Propofol group.

The main effect of time was significant (F = 134.92, P < 0.001, partial η² = 0.0391), indicating that the administration of the study medication resulted in a significant decrease in SBP for both groups. A significant main effect of group was observed (F = 5.009, P = 0.026, partial η² = 0.023), indicating that participants in the propofol group exhibited lower SBP compared to those in the ciprofol group across all time points (Table 4). Notably, the ciprofol group exhibited significantly less decrease in blood pressure compared to the propofol group (Fig 3A–3C; P < 0.05). Within 2 minutes post-intubation, patients in both groups experienced a transient HR increase, with those in the ciprofol group showing a significantly smaller increase than those in the propofol group (Fig 3D).

**Table 4:** Main effects of group and time and their interaction effect on hemodynamic parameters.

Outcome measure	Effects	F	P-value	Partial η²
SBP	Group	5.009	0.026^†^	0.023
	Time	134.92	<0.001^†^	0.391
	Group*Time	4.168	<0.001^†^	0.019
DBP	Group	1.267	0.262	0.006
	Time	87.579	<0.001^†^	0.294
	Group*Time	1.497	0.173	0.007
MAP	Group	3.498	0.063	0.016
	Time	108.16	<0.001^†^	0.34
	Group*Time	2.494	0.022^†^	0.012
HR	Group	2.846	0.093	0.013
	Time	72.618	<0.001^†^	0.257
	Group*Time	4.058	0.002^†^	0.019

† The main effects of Group and Time and their interaction effect at significance level <0.05.

SBP: systolic blood pressure, DBP: diastolic blood pressure, MAP: mean arterial pressure, HR: heart rate.

Adverse events encountered during the induction of anesthesia are detailed in Table 4. Patients in the ciprofol group exhibited a significantly lower overall rate of adverse events compared to those in the propofol group (25.47% vs. 89.62%, P < 0.001). The incidence of injection pain was significantly reduced in the ciprofol group (0.94% vs. 25.58%, P < 0.001). Additionally, when compared to the propofol group, the ciprofol group showed significantly lower incidences of tachycardia and hypotension (6.60% vs. 19.81%, P = 0.005; 14.15% vs. 37.74%, P < 0.001) (Table 5). Notably, all observed adverse events were mild, with no cases of severe adverse events.

**Table 5:** Incidence of adverse events during induction.

Variable	Ciprofol group (n = 106)	Propofol group (n = 106)	p-value
Injection-site pain, n (%)	1 (0.94%)	25 (25.58%)	0.000^*^
Allergic reaction, n (%)	1 (0.94%)	2 (1.89%)	0.561
Bradycardia, n (%)	2 (1.89%)	3 (2.83%)	0.651
Tachycardia, n (%)	7 (6.60%)	21 (19.81%)	0.005^*^
Hypotension, n (%)	15 (14.15%)	38 (37.74%)	0.000^*^
Hypertension, n (%)	1 (0.94%)	3 (2.83%)	0.313
Hypoxemia, n (%)	0	0	
Intubation response, n (%)			
Swallowing, n (%)	0	0	
Cough, n (%)	0	0	
Body movement, n (%)	0	0	
Tears, n (%)	0	2 (1.89%)	0.155
Bronchospasm, n (%)	0	1 (0.94%)	0.316
Total number of adverse events, n (%)	27 (25.47%)	95 (89.62%)	0.000^*^

* P < 0.05 vs. Propofol group

## Discussion

The present study evaluated the safety and effectiveness of ciprofol as a general anesthesia induction agent for individuals with obesity undergoing LSG. Given the unique challenges obesity presents in surgical procedures, such as altered pharmacokinetics and increased complication risks, selecting an appropriate anesthesia induction agent is vital for optimal surgical conditions and minimizing obesity-related risks.

We compared the efficacy of ciprofol at a dosage of 0.5 mg/kg to propofol at a dosage of 2.5 mg/kg regarding anesthesia induction success rate when combined with standard opioid-benzodiazepine coadministration. Notably, our findings demonstrated that ciprofol at 0.5 mg/kg was non-inferior to propofol at 2.5 mg/kg, with both achieving 100% success in anesthesia induction. This finding is consistent with pharmacokinetic data suggesting that ciprofol’s potency is approximately 4–5 times that of propofol, thus justifying the dose ratio selected for comparison [18]. Importantly, these results reflect real-world clinical practice, where induction agents are routinely administered synergistically with opioids and benzodiazepines to optimize hemodynamic stability and patient tolerance. The 100% success rate reflects the protocol’s pragmatic design (allowing top-up administration) rather than a lack of differentiation between agents.

While the time for successful anesthesia induction and eyelash reflex disappearance was slightly longer in the ciprofol group, both agents effectively induced sedation rapidly, typically within a minute. Furthermore, our findings highlighted ciprofol’s potentially stronger GABA A receptor binding activity compared to that of propofol, given the significantly lower dose required to induce anesthesia (ciprofol: 0.5 mg/kg vs. propofol: 2.5 mg/kg), aligning with previous research [7].

BIS values, used to assess anesthetic state and sedation level, showed similar patterns between the groups in the initial 20 minutes post-drug administration. However, differences emerged in late post-induction phase BIS profiles, with ciprofol exhibiting lower average BIS values from 10 to 20 minutes post-administration compared to propofol. This suggests a more potent sedative effect of ciprofol at this dosage. Compared with the propofol group, more patients in the ciprofol group had BIS values ranging from 40 to 60 during this period. This suggests that ciprofol maintains an appropriate level of anesthesia depth.

The occurrence of injection pain during medication administration is a frequently observed adverse reaction in patients receiving propofol anesthesia. We found a significantly lower incidence of injection-related pain with ciprofol compared with propofol (0.94% vs. 25.58%). Ciprofol, an isomer of propofol, includes a cyclopropyl moiety in its chemical structure, which enhances its pharmacological and physicochemical properties, leading to diminished injection pain. The incidence of injection pain observed in the propofol group (25.58%) was notably lower than previously reported [19], which may be attributed to the preoperative administration of sufentanil.

Hypotension is another adverse effect commonly associated with propofol administration, which can increase the risk of myocardial and renal injury when SBP drops below 90 mmHg [8]. Moreover, reducing SBP by 41 − 50 mmHg from baseline for >5 minutes triples the risk of myocardial infarction, while maintaining MAP below 80 mmHg for over 10 minutes can increase mortality rates [8]. The risk increases as the duration lengthens, and the MAP decreases [20,21]. In the present study, both groups experienced decreased blood pressure within 3 minutes of medication administration. However, the ciprofol group showed a significantly smaller decrease in blood pressure and a significantly lower occurrence of hypotension requiring treatment compared to the propofol group. The findings indicate that ciprofol offers greater benefits than propofol in maintaining hemodynamic stability for patients undergoing anesthesia or sedation procedures. By minimizing blood pressure reductions and the incidence of hypotension, interventions or treatment, such as fluid resuscitation or vasopressor administration, could be potentially avoided. Stable hemodynamics during medical procedures are crucial to prevent complications, such as organ damage, thereby reducing overall morbidity and mortality.

The ciprofol group also exhibited lower overall rates of adverse events compared to those in the propofol group. Adverse events in both groups were effectively managed with minimal or short-term medication treatments without significant negative outcomes. These results indicate that ciprofol may be a preferable alternative to propofol due to its lower adverse event rates and effective management of complications.

The present study had some limitations, including its single-center design, thus warranting further large-scale multicenter studies to confirm the findings. The protocol-specific use of midazolam and sufentanil co-administration, which may not reflect clinical practice in settings where alternative agents or dosages are employed. The allowance for supplemental doses may have contributed to the uniform success rate, potentially obscuring differences in initial dose efficacy. However, this approach mirrors clinical practice and ensures patient safety, which remains paramount in anesthesia trials. Moreover, the utilization of ciprofol during anesthesia maintenance was not investigated. Therefore, additional research should be undertaken to explore both the induction and maintenance of anesthesia.

## Conclusion

Ciprofol has demonstrated non-inferiority to propofol regarding the success rates of anesthesia induction and offers a favorable sedative effect with reduced adverse events, particularly in hemodynamic stability and injection pain. These findings position ciprofol as a promising alternative to propofol in patients with obesity undergoing LSG. Further research and clinical trials are necessary to validate these findings and explore additional benefits associated with the use of ciprofol in this specific demographic.

## Supporting information

S1 File Inclusion and exclusion criteria. (DOCX)

S2 File MOAA/S Responsiveness Scale. (DOCX)

S3 File Datasets. (XLSX)

S4 File CONSORT 2010 checklist of information to include when reporting a randomised trial*. (DOC)

S5 File Protocol-Chinese version. (DOCX)

S6 File Protocol-English version. (DOCX)
