# Supplementary material for: Efficacy and safety of ciprofol for the induction of general anesthesia in patients with obesity undergoing laparoscopic sleeve gastrectomy: A double-blind randomized, controlled study
Source: PLoS One. 2025 Jul 24;20(7):e0329005. doi: 10.1371/journal.pone.0329005 (PMC12289008; doi:10.1371/journal.pone.0329005)
Supplement: S1 File — (DOCX) [file pone.0329005.s001.docx]

**Supplemental File 1.** Inclusion and exclusion criteria.

***Inclusion criteria***

*Subjects who met all of the following criteria were enrolled in the trial*

1. Subjects scheduled for laparoscopic sleeve gastrectomy under general anesthesia.

2. 18 < age ≤ 65, regardless of gender.

3. Subjects with ASA score I-III.

4. Subjects with a body mass index (BMI)≥35kg/m2.

5. Subjects with blood pressure between 90-140/50-90 mmHg (inclusive); heart rate between 60-100 bpm (inclusive); body temperature between 35.4-37.5°C (inclusive); respiratory rate between 12-20 breaths per min (inclusive); SpO2 when inhaling≥92%.

6. Subjects with normal results of physical examination, laboratory tests, (routine blood/urine, blood biochemistry (including hepatic function, renal function, blood glucose, and electrolytes such as Na, K and Mg), and blood coagulation), 12-lead ECG, and abdominal ultrasonography, or abnormalities considered by the investigators to be clinically insignificant; no potential significant difficult airway problems (modified Mallampati score of I-II).

7. No previous history of primary diseases in major organs, such as the liver, kidneys, digestive tract, and blood; no history of malignant hyperthermia or other hereditary disorders; no history of mental/neurological disorders; no history of epilepsy; no contraindications for deep sedation/general anesthesia; no clinically significant history of anesthesia accidents.

8. Subjects understood the procedures and methods employed in the trial and were willing to sign informed consent forms and complete the trial in strict accordance with the designated protocol.

***Exclusive Criteria***

*Subjects who met all of the following criteria were excluded from the trial*

1. Patients who refused to participate in the study.
2. A history of allergy or hypersensitivity to the study drugs or its excipients which would have been used in the study.
3. Subjects who had a history or evidence of any of the following diseases prior to screening/administration:
4. A history of cardiovascular disease, such as postural hypotension, serious heart valve disease, severe arrhythmia, heart failure, Adams-stokes syndrome, unstable angina pectoris; myocardial infarction within 6 months before screening, tachycardia/bradycardia requiring medications, third degree atrioventricular conductive block or a QTcF interval≥450 ms (per Fridericia’s correction formula).
5. Subjects screened for a history of bronchospasm that required treatment within the first 3 months; or subjects who developed acute respiratory tract infections, with symptoms of fever, wheezing, nasal congestion or cough within 1 week prior to baseline.
6. A history of psychiatric or neurological disorders, such as brain injury, possible intracranial hypertension, cerebral aneurysm, cerebrovascular accident history and central nervous system diseases, mental system diseases (schizophrenia, mania, insanity, etc.) and long-term history of taking psychotropic drugs.
7. *Laboratory results meeting any of the following during screening/at baseline:*
8. Abnormal liver function (ALT or AST ≥ 2.5 times the upper limit of normal value, TBIL ≥ 1.5 times the upper limit of normal value), abnormal renal function (urea ≥ 1.5 times the upper limit of normal value, serum creatinine> the upper limit of normal value, or dialysis treatment within 28 days before operation), obvious abnormal coagulation function, anemia or thrombocytopenia (HB ≤ 90g/l, PLT ≤ 80 × 109/L).
9. A positive result for any of the following markers: HBsAg, HCV-Ab, HIV-Ab, and Tp-Ab.
10. Other relevant factors:
11. Unregulated diabetes mellitus and hypertension. (Fasting blood glucose ≥11.1mmol/L during screening, and/or random blood glucose ≥ 13.6mmol/L; SBP≥160mmHg and/or DBP≥100 mmHg during the screening period).
12. Subjects who received propofol, other sedatives/anesthetics and/or opioid analgesics within 1 week prior to enrollment.
13. Had a history of drug abuse within 2 years before the screening period or had a positive result of drug screening in urine at baseline.
14. A history of alcoholism within 3 months prior to screening; alcoholism defined as an average of>2 units of alcohol per day (1 unit=360 mL beer or 45 mL liquor with 40% alcohol or 150 mL wine), or had a positive alcohol breath test result at baseline.
15. Subjects who smoked>5 cigarettes per day and had a total of>60 cigarettes within 3 months prior to screening.
16. Subjects who developed clinically significant acute disease (determined by the investigators), such as infection (respiratory tract, CNS infections, septicemia, myocarditis or endocarditis), within 2 weeks prior to screening.
17. The subjects who were judged to have difficulty in respiratory management were rated as grade IV by Modified Mallampati Score.
18. Pregnant or lactating women or subjects with birth plan within 6 months (including men).
19. Those who participated in any clinical trial as subjects within 3 months prior to screening.
20. Communication difficulties.
21. Subjects judged by the investigators to be unsuitable for participating in the trial for any reason.
