# Supplementary material for: Efficacy and safety of ciprofol for the induction of general anesthesia in patients with obesity undergoing laparoscopic sleeve gastrectomy: A double-blind randomized, controlled study
Source: PLoS One. 2025 Jul 24;20(7):e0329005. doi: 10.1371/journal.pone.0329005 (PMC12289008; doi:10.1371/journal.pone.0329005)
Supplement: S2 File — (DOCX) [file pone.0329005.s002.docx]

**Supplementary File 2.** MOAA/S Responsiveness Scale

| Responsiveness | **Score** | **Classification** |
| --- | --- | --- |
| Responds readily to name spoken in normal tone | 5 | alert |
| Lethargic response to name spoken in normal tone | 4 | sedation |
| Responds only after name is called loudly and/or repeatedly | 3 | sedation |
| Responds only after mild prodding or shaking | 2 | sedation |
| Responds only after painful trapezius squeeze | 1 | loss of consciousness |
| Does not respond to painful trapezius squeeze | 0 | loss of consciousness |

MOAA/S: Modified Observer’s Assessment of Alertness/Sedation.
