# Supplementary material for: Efficacy and safety of ciprofol for the induction of general anesthesia in patients with obesity undergoing laparoscopic sleeve gastrectomy: A double-blind randomized, controlled study
Source: PLoS One. 2025 Jul 24;20(7):e0329005. doi: 10.1371/journal.pone.0329005 (PMC12289008; doi:10.1371/journal.pone.0329005)
Supplement: S5 File — (DOCX) [file pone.0329005.s005.docx]

成都市医学科研课题申报书

| 流水号 | 202204111826 |
| --- | --- |
| 课题编号 | 2022063 |
| 课题名称 | 环泊酚应用于腹腔镜袖状胃切除术肥胖患者麻醉诱导的有效性与安全性研究 |
| 申报单位 | 成都市第三人民医院 |
| 课题负责人 | 池小薇 |
| 研究起止年限 | 2022 至 2024 |
| 通讯地址 | 成都市青羊区青龙街82号成都市第三人民医院麻醉科 |
| 联系电话 | 13708085545 |
| 申报日期 | 2022-03-11 |

成都市卫生健康委员会

| **一、研究目的和主要研究内容。** |
| --- |
| **研究目的：**分析和研究环泊酚用于腹腔镜袖状胃切除术肥胖患者的全身麻醉诱导的有效性和安全性。拟选取2022年12月01日起至2024年11月30日，于笔者所在医院行腹腔镜袖状胃切除术的肥胖患者，采用随机、单盲、平行、丙泊酚注射液阳性对照的研究方法，将两组患者的主要观察指标、次要观察指标、安全性评价指标、不良事件等进行对比。评价环泊酚用于腹腔镜袖状胃切除术肥胖患者中全身麻醉诱导的有效性和安全性。  **主要研究内容：**  **1.主要观察指标：**环泊酚麻醉诱导成功的比例。成功的标准为同时满足以下2项要求：1）给予研究药物后（至多允许追加2次）达到诱导成功［即改良研究者清醒/镇静评分（MOAA/S）≤ 1］；2）未使用替代麻醉药物。  **2.次要观察指标：**研究药物开始给药至受试者意识消失的时间；睫毛反射消失的时间；各监测点BIS值的变化；研究药物和补救药物使用情况；麻醉诱导满意度。  **3.安全性评价指标：**不良事件；插管反应率；生命体征( 血压(收缩压/舒张压)、心率、呼吸频率、SpO2)；心电图；给药部位观察；实验室检查(血常规、血生化、尿常规、凝血功能)；监测时间点中收缩压维持在≥80和＜150 mmHg范围内的时间点比例。注射痛评价。  **4.需要重点关注的不良事件：**麻醉过程中血压降低的发生率；麻醉诱导过程中需要治疗的血压降低发生率；呼吸抑制发生率；麻醉过深或麻醉过浅发生率；术中知晓的发生率。 |
| 二、研究背景和依据（国内外研究现状、发展趋势、必要性、新颖性、创新点、研究意义）。 |
| 随着舒适化医疗及加速康复外科(enhanced recovery after surgery，ERAS)的逐步发展，医护人员和患者都追求更加舒适化的诊疗，为进一步提高手术患者就医舒适度，优化围术期的麻醉管理便尤为重要[1],从而最小化不良反应，提高康复质量[1]。        在临床实践中，肥胖患者在生理及机体组织上的改变，可影响大多数药物的药代动力学特性[2]。丙泊酚已被广泛应用于肥胖患者的全身麻醉中，高脂溶性的丙泊酚会增加肥胖患者的静观分布容积，延长消除半衰期[3]。有研究结果显示，与年龄、身高和性别均相同的正常体重患者相比，肥胖患者的脂肪和瘦体重（LBW）均有所增加，其每千克总体重（TBW）中脂肪的增加程度更为显著，而每千克TBW中的LBW减轻。脂肪的血流显著少于瘦组织（如心脏、脑等），且心输出量主要分布于瘦组织，其与LBW呈正比[4]，是药物早期分布的一个重要动力学参数。因此，肥胖患者行全身麻醉时的丙泊酚诱导剂量应按LBW计算。瘦体重（LBW）也称去脂体重，肌肉是其中的主要部分。男性LBW=9.27×103×TBW/(6.68×103+216×BMI)；女性LBW=9.27×103×TBW/(8.78×103+244×BMI) [3-5]；BMI为体重指数。        丙泊酚具有起效快，作用时间短等特点，它能在大脑和终端代谢间进行快速的再分配，它主要通过CYP2B6酶在肝脏形成葡萄糖醛酸络合物或氧化物代谢。丙泊酚在用于麻醉时虽然被认为很有效，但是却有着安全性和耐受性方面的问题：(1)在使用时会对心血管系统产生作用并有呼吸抑制的副作用，手术时需要麻醉医师的参与；(2)注射时有疼痛；(3)制剂包装有细菌污染的风险；(4)不适合长期使用。        环泊酚是中国首个自主化合物创新静脉麻醉药，具有如下优点：1）起效快速，苏醒迅速；2）更少剂量，起效高，镇静优；3）更少呼吸抑制，呼吸抑制风险降低逾60%；4）更少注射痛，注射痛发生率仅为丙泊酚的1/10。环泊酚I期临床试验发现：该药物起效迅速，作用时间短；无注射痛；无显著生命体征变化；呼吸暂停情况与丙泊酚类似；表现出剂量依赖性的镇静/麻醉效应；效价约为异丙酚4～5倍；T1/2 keo:0.59～3.22min；未探索到最大耐受剂量(MTD)[6]。中国IIa期结肠镜研究设计主要用于探索环泊酚在诊断性结肠镜检查受试者中镇静/麻醉的最大耐受剂量(MTD)和后续研究的推荐剂量，研究结果发现环泊酚0.4～0.5 mg/kg 与丙泊酚2.0mg/kg组相当, 其效价为丙泊酚的4～5倍，与丙泊酚组相比，环泊酚组患者需要进行气道管理的比例更低。中国结肠/胃镜III期研究设计评价与丙泊酚相比，环泊酚乳状注射液在结肠镜和/或胃镜诊疗受试者中镇静/麻醉的有效性，研究结果发现：1）环泊酚与丙泊酚相比诱导成功时长无差异；2）环泊酚组较丙泊酚组的追加次数少，需要追加的人群比例更低，结肠镜诊疗过程中保持清醒的人比例也更少；3）由于环泊酚0.4 mg/kg的药效强于丙泊酚1.5 mg/kg，导致该剂量下的清醒时间和离室的时间比丙泊酚略长；4）环泊酚组注射痛发生率显著低于丙泊酚，与环泊酚的I-II期结果保持一致；5）环泊酚组对结肠/胃镜退出的知晓比例显著低于丙泊酚组，受试者平均满意度显著优于丙泊酚；6）环泊酚组麻醉科医师对受试者舒适度的评价显著优于丙泊酚组；7）环泊酚组受试者对内镜退出的记忆比例显著低于丙泊酚组。        目前的各项临床研究数据均来自体重指数（BMI）≥18且≤30kg/m2的患者，尚无针对BMI＞30 kg/m2的相关研究。本研究拟选择全身麻醉下行腹腔镜袖状胃切除术的肥胖患者，采用随机、单盲、平行、丙泊酚注射液阳性对照的研究方法，在腹腔镜袖状胃切除术中评估环泊酚用于全身麻醉诱导期间的有效性和安全性。    参考文献：  [1] 中华医师协会麻醉学医师分会.促进术后康复的麻醉管理专家共识[J].中华麻醉学杂志，2015,35(2):141-146.  [2] LEMMENS H J. Perioperative pharmacology in morbid obesity[J].Curr Opin Anaesthesiol,2010,23(4):485-491.  [3] TACHIBANA N, NIIYAMA Y, YAMAKAGE M. Evaluation of bias in predicted and measured propofol concentrations during target-controlled infusions in obese Japanese patients: an open-label comparative study[J].Eur J Anaesthesiol,2014,31(12):701-707.  [4] LEMMENS H J,BRODSKY J B,BERNSTEIN D P. Estimating ideal body weight-a new formula[J].Obes Surg,2005,15(7):1082-1083.  [5] DOMI R,LAHO H. Anesthetic challenges in the obese patient[J].J Anaesth,2012,26(5):758-765.  [6] Linlin Qin, etal. Design, synthesis, and evaluation of novel 2,6-Disubstituted Phenol Derivatives as general anesthestics [J].J Med Chem.2017,60,3606-3617. |
| 三、研究方法、技术路线、计划进度和阶段目标。 |
| **研究设计类型：**随机、双盲、丙泊酚平行对照  **随机方法：**本研究采用简单随机且隐藏随机方案。研究小组统计学专家在SPSS25.0软件上以20221001为固定数字产生212个随机数字，并随机分为两组。把随机方案装入不透光的信封，每个信封封面写有一个筛选号，为进入筛选的受试者的顺序号。信封里有该受试患者的随机号及分组情况。  **盲法：**试验对受试者、手术者、和统计人员设盲。在整个研究过程中（包括随访）不能对受试患者和受试患者家属揭盲。  **研究中心：**成都市第三人民医院  **样本量计算：**本研究拟对实验组和对照组的有效性和安全性进行比较。因此采用完全随机设计的两独立样本率检验样本量估算公式对样本量进行估算。评价麻醉诱导成功需同时满足以下两个条件：1.给予研究药物后（至多允许追加2次）达到诱导成功［即改良研究者清醒/镇静评分（MOAA/S）≤ 1］；2.未使用替代麻醉药物。评价安全性指标主要有：生命体征、插管反应率、不良事件、心电图、实验室指标、注射筒评价。根据文献非肥胖患者研究发现对照组丙泊酚诱导药物相关不良事件发生率为70.5%。试验组环泊酚诱导药物相关不良事件发生率为64.8%。假设检验的Ⅰ类错误为0.05，检验效能1-β为80%，两组样本量比例为1:1，根据样本量双侧检验以及增加10%的患者退出、剔除、失访等情况，计算样本量约为212例，每组106例。  **入选标准：**  （1）18周岁＜年龄≤65周岁，性别不限；  （2）ASA 分级为Ⅰ～Ⅲ级；  （3）BMI≥35kg/m2；  （4）需要在全身麻醉下进行腹腔镜袖状胃切除术的肥胖患者；  （5）受试者自愿参加本试验，并签署知情同意书。  **排除标准：**  （1）有全身麻醉禁忌症者；  （2）伴有感染性心脏疾病如心肌炎或心内膜炎，败血症；  （3）颅脑损伤、可能存在颅内高压、脑动脉瘤、脑血管意外史及患有中枢神经系统疾病者：精神系统疾病(精神分裂症、躁狂症、精神错乱等)及长期服用精神类药物史，或患有其他妨碍测量BIS值疾病者；  （4）急性心力衰竭，不稳定型心绞痛，筛选前6个月内发生心肌梗死，静息心电图心率≤50次/分，三度房室传导阻滞等严重心律失常，严重的心脏瓣膜疾病，QTC：男性≥450ms,女性≥470ms；  （5）肝肾功能异常(ALT或AST≥2.5倍正常值上限、TBIL≥1.5倍正常值上限)，肾功能异常(BUN或Urea≥1.5倍正常值上限、Cr>正常值上限、或手术前28天内进行过透析治疗)，或者凝血功能明显异常者(PT/APTT/TT高于正常值上限)，贫血或者血小板减少(Hb≤90g/L、PLT≤80×109/L)；  （6）血压未获满意控制者(筛选期坐位收缩压SBP≥160mmHg，和/或舒张压DBP≥100 mmHg)；  （7）筛选期坐位收缩压SBP≤90mmHg；  （8）血糖未获满意控制的糖尿病受试者(筛选期空腹血糖≥11.1mmol/L，和/或随机血糖≥13.6 mmol/L)；  （9）筛选期开始前2年内有吸毒史和酗酒史，酗酒定义为定期饮酒超过14次/周(1次=150 ml葡萄酒或360 ml啤酒或45 ml烈酒)；  （10）被判定为呼吸道管理有困难的受试者，改良马氏评分为IV级；  （11）已知或怀疑对研究药物各种组分或其他苯二氮卓类药物、阿片类药物、丙泊酚、肌松药等过敏或禁忌者；  （12）妊娠或哺乳期女性或6个月内有生育计划的受试者(包括男性)；  （13）入选前3个月内作为受试者参加过任何临床试验者；  （14）经研究者判定不适合入选的其它情况。  **治疗方案：**  试验药：环泊酚注射液  剂型：注射剂  规格：20ml:50mg  用法用量：使用微量泵静脉输注，麻醉诱导剂量0.4-0.5mg/kg,给药30s (±5s)。男性去脂体重LBW=9.27×103×TBW/(6.68×103+216×BMI)；女性LBW=9.27×103×TBW/(8.78×103+244×BMI)；BMI为体重指数。TBW为总体重。  阳性对照药：丙泊酚注射液  剂型：注射剂  规格: 20ml: 200mg  用法用量:使用微量泵静脉输注，麻醉诱导剂量2-2.5mg/kg，给药30s (±5s)。男性去脂体重LBW=9.27×103×TBW/(6.68×103+216×BMI)；女性LBW=9.27×103×TBW/(8.78×103+244×BMI)；BMI为体重指数。TBW为总体重。  **主要观察指标**  环泊酚麻醉诱导成功的比例，成功的标准为同时满足以下2项要求：1）给予研究药物后（至多允许追加2次）达到诱导成功［即改良研究者清醒/镇静评分（MOAA/S）≤ 1］；2）未使用替代麻醉药物。  **次要观察指标**  研究药物开始给药至受试者意识消失的时间；  睫毛反射消失的时间；  患者麻醉诱导期间血流动力学指标的变化；  各监测点BIS值的变化；  研究药物和补救药物使用情况；  麻醉诱导满意度。  **安全性评价指标**  不良事件；  插管反应率；  生命体征( 血压(收缩压/舒张压)、心率、呼吸频率、SpO2)；  心电图；  给药部位观察；  实验室检查(血常规、血生化、尿常规、凝血功能)；  监测时间点中收缩压维持在≥80和＜150 mmHg范围内的时间点比例。  注射痛评价。  **需要重点关注的不良事件**  麻醉过程中血压降低的发生率；  麻醉过程中需要治疗的血压降低发生率；  呼吸抑制发生率；  麻醉过深或麻醉过浅发生率；  术中知晓的发生率；  给药部位情况。    **设计方案**  1 筛选期(D-7到D0天随机入组前)     实施研究药物治疗的当天定义D0天，在研究药物随机入组前，患者应完成以下检查和信息收集。     1)签署知情同意书，收集患者人口学资料；     2)5年内的重大既往病史及手术史(或5年以外但研究者认为需要记录的)、过敏史、药物或酒精滥用史、伴随疾病、合并用药、生育计划等；     3)体格检查：一般检查(含ASA分级和改良马氏评分)；     4)体重：采集患者总体重（TBW）(四舍五入保留整数)，计算去脂体重（LBW），男性LBW=9.27×103×TBW/(6.68×103+216×BMI)；女性LBW=9.27×103×TBW/(8.78×103+244×BMI)；BMI为体重指数；麻醉诱导时以LBW。     5)生命体征：心率、呼吸频率、SpO2和血压(收缩压/舒张压)；     6)实验室检查：血常规、血生化、尿常规、妊娠测试(血妊娠或尿妊娠)、凝血功能；     7)十二导联心电图检查；     8)审核患者入选与排除标准；     9)合并用药：需记录随机分组前7天内合并用药。  2 治疗期(D0)  2.1 术前准备(D0)     1)再次确认患者入选与排除标准；     2)确定入选患者，经随机系统分配，随机入组；     3)安放监测设备完毕后，采集基线值，包括心率、血压(收缩压舒张压)、呼吸频率、SpO2、BIS；     4)开放静脉通路；     5)开始面罩式吸氧，氧流量维持在2～4 L/min；     6)记录合并用药/治疗；  2.2 麻醉诱导(D0)     1)静脉给予环泊酚或丙泊酚前2min(+1min)静脉缓慢推注咪达唑仑0.04mg/kg 15s推注完成、枸橼酸舒芬太尼注射液0.3μg/kg 30s推注完成；     2)研究者开始使用微量泵静脉输注环泊酚或丙泊酚注射液：试验组环泊酚注射液诱导量0.4-0.5mg/kg，给药时间30s (±5s)；如果给药结束后1min±10s未丧失意识(LOC) (MOAA/S 评分为＞1分)，允许追加环泊酚，追加剂量为50%初始剂量，10s完成推注；继续观察1min±10s，若MOAA/S 评分仍＞1分，则进行第二次追加，剂量同上；继续观察1min±10s，若MOAA/S 评分仍＞1分，则认为诱导失败，换用丙泊酚替代，剂量由研究者决定。对照组丙泊酚注射液麻醉诱导剂量2-2.5mg/kg，给药时间30s (±5s)，评估是否追加诱导剂量方法同上 ；     3)受试者LOS后，静脉注射肌松剂罗库溴铵注射液，给药剂量为0.6 mg/kg 15s推注完成，给予辅助通气，待达到满意的肌松状态后，麻醉医生进行气管插管，气管插管后行机械通气；     4) MOAA/S评分：诱导开始时记录1次，诱导成功时(MOAA/S评分为≤1分)记录1次，需具体到秒，；     5）记录给药过程是否有注射痛；     6）记录研究药物开始给药至患者意识消失的时间；     7）记录麻醉诱导过程中血流动力学指标的变化；     8）记录不良事件和合并用药/治疗。  2.3 麻醉维持 (D0)：由麻醉医生决定麻醉维持药物的选择  2.4 手术结束后(D0)：转运至麻醉复苏室（PACU）    **技术路线**  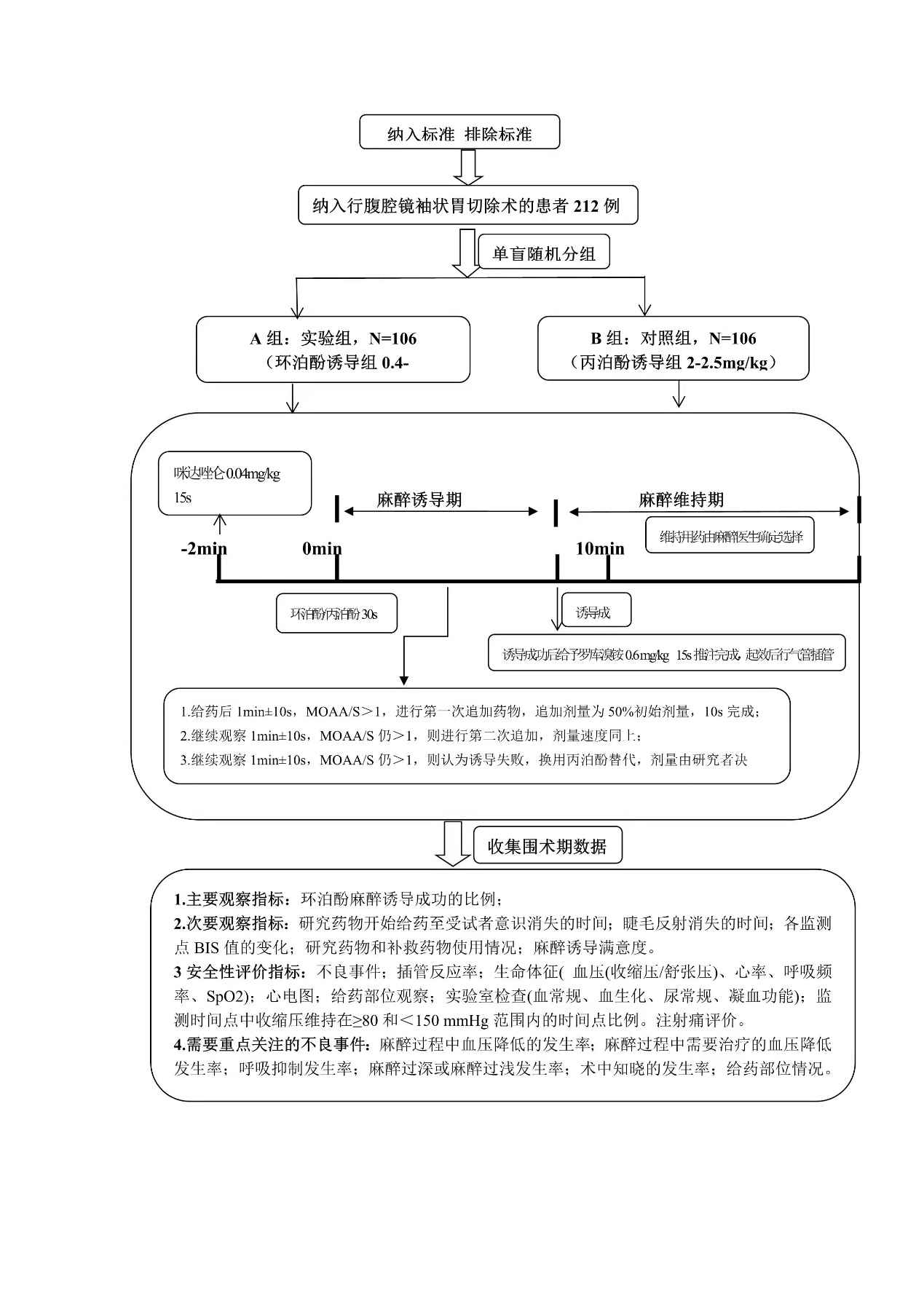  **计划进度和阶段目标**：  第一阶段（2022.12.01-2023.05.31）:预期顺利完成临床研究的实施，收集完数据，待分析  第二阶段（2023.06.01-2023.12.31）:预期顺利按期进行数据的整理、统计、分析  第三阶段（2024.01.01-2024.12.01）:预期完成论文撰写，完成1—2篇课题论著，发表SCI/Medline级别文章1篇，中文核心期刊1篇。 |
| 四、已具备的条件（包括前期研究工作、实验室设备、实验动物和动物实现、信息资料等）。 |
| 1.研究基础（前期研究工作积累和已取得的研究工作成绩）  1.1 与本项目相关的前期研究工作积累        本单位减重代谢手术开展10余年，并率先于西南地区创建肥胖于代谢疾病中心，已经成为西南地区代谢减重手术量最大的中心。代谢减重麻醉团队力量厚实，从而为本研究提供研究基础。减重麻醉团队实力雄厚，具备完善的术前术中术后手术麻醉记录系统，具备完善的术后随访机制。课题组成员均有丰富的临床经验和科研研究经验，均能完成与本课题有关的临床观察、数据收集。主要研究人员配备合理，已形成研究梯队，有较强的理论水平和研究能力，可顺利解决项目实施过程中关键问题，对项目实施提供了必要的技术保障，可以完成相关实验任务。  1.2 已取得的前期研究工作        前期我们将环泊酚应用于无痛胃肠镜诊疗，得出环泊酚组的诊疗成功率均为 100%，说明 环泊酚与丙泊酚相当，达到主要终点。环泊酚与丙泊酚药物追加次数分别为(0.5±0.85)次和 (0.7±0.71)次（P=0.052)（见图一）。环泊酚与丙泊酚相比诱导成功时长无差异，由于环泊酚 0.4 mg/kg 的药效强于丙泊酚 1.5 mg/kg，导致该剂量下清醒时间和苏醒时间比丙泊酚略长（见图二）。环泊酚组呼吸系统相关药物相关不良事件（呼吸抑制、呼吸暂停、缺氧）发生率低于 丙泊酚组，与前期研究结果一致，按例次计算 P＜0.05。结肠镜中环泊酚呼吸暂停、缺氧的持续时间均短于丙泊酚（见图三）。  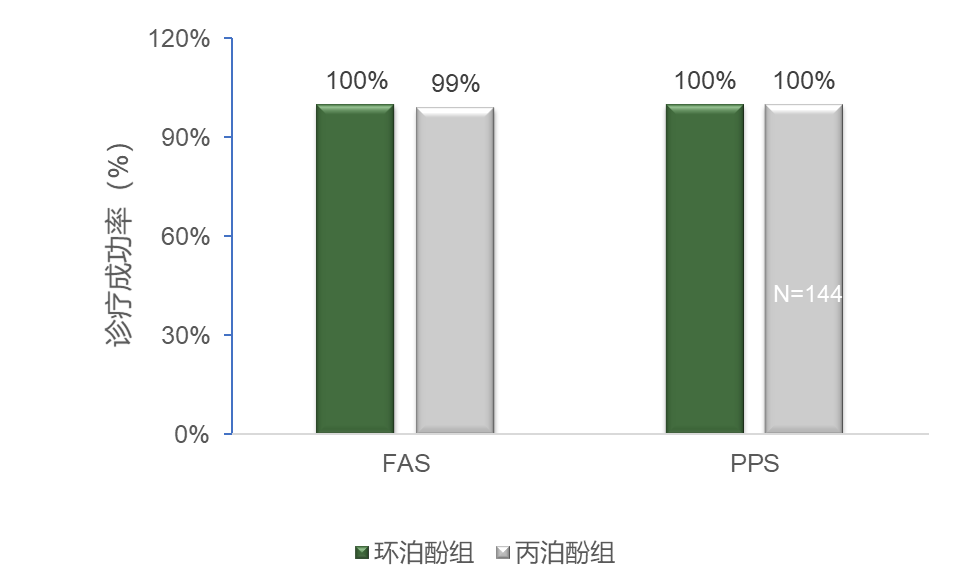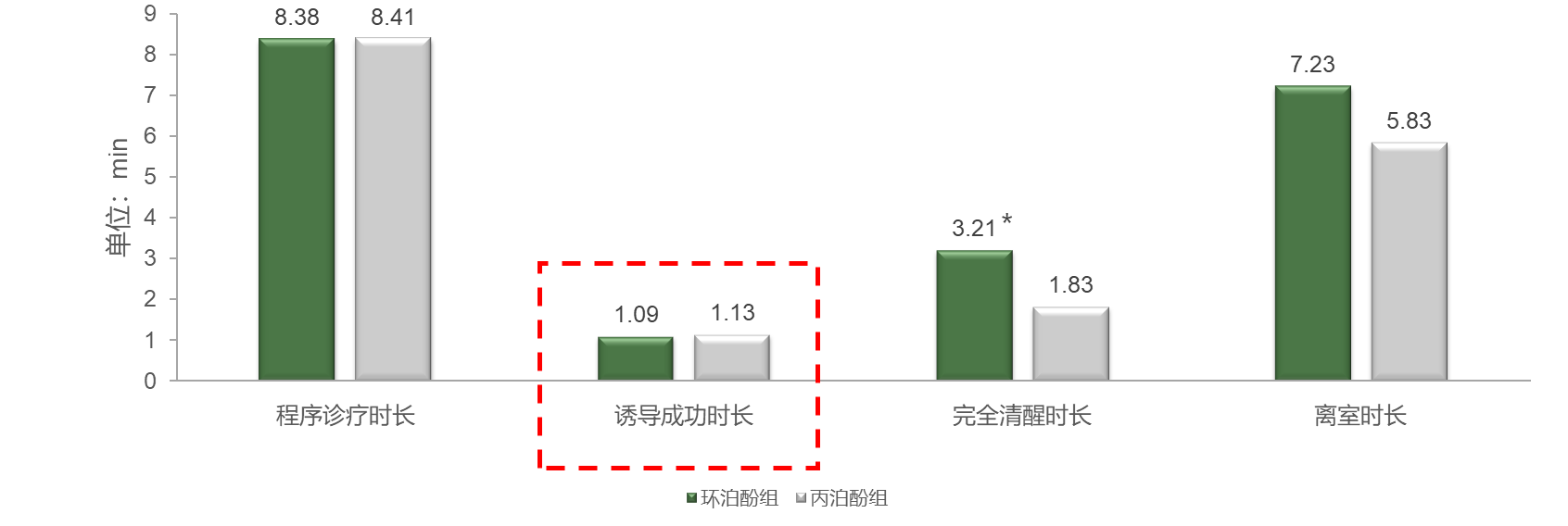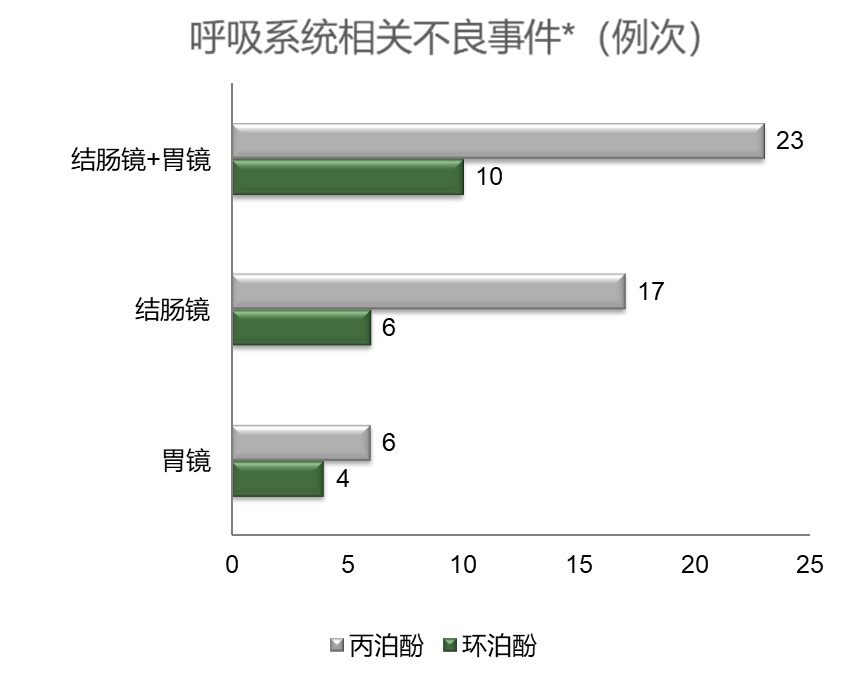 |

| 十二、伦理证明。 |
| --- |
|  |
| 十三、查新检索。 |
|  |
| 审查意见 |
|  |
|  |
