# Supplementary material for: Efficacy and safety of ciprofol for the induction of general anesthesia in patients with obesity undergoing laparoscopic sleeve gastrectomy: A double-blind randomized, controlled study
Source: PLoS One. 2025 Jul 24;20(7):e0329005. doi: 10.1371/journal.pone.0329005 (PMC12289008; doi:10.1371/journal.pone.0329005)
Supplement: S6 File — (DOCX) [file pone.0329005.s006.docx]

**Medical Research Project of Chengdu Medical Association**

| Serial number: | 202204111826 |
| --- | --- |
| Grant number: | 2022063 |
| Title: | Efficacy and safety of ciprofol for the induction of general anesthesia in patients with obesity undergoing laparoscopic sleeve gastrectomy |
| Application  department: | Department Of Anesthesiology, The Third People’s Hospital of Chengdu |
| Applicant: | Xiaowei Chi |
| Date of research: | 2022 to 2024 |
| Contact address: | Department of Anesthesiology, Third People’s Hospital of Chengdu Affiliated to Southwest Jiaotong University  No. 82, Qinglong Street, Chengdu, Sichuan, China, IN 610031 |
| Telephone number: | 13708085545 |
| Date of application: | 2022-03-11 |

成都市卫生健康委员会

| **1、Research purpose and main research content** |
| --- |
| **Research purpose：**To evaluate the efficacy and safety of ciprofol for the induction of general anesthesia in obese patients undergoing laparoscopic sleeve gastrectomy. A randomized, double-blind, parallel, propofol injection positive control study will be conducted to select obese patients who will undergo laparoscopic sleeve gastrectomy in the author's hospital. To evaluate the efficacy and safety, the main observation index, secondary observation indexes, safety evaluation indexes, and the incidence of postoperative adverse reactions will be recorded and compared between the two groups.  **main research content:**  **1. *Primary outcome*：**The primary endpoint was the success rate of general anesthesia induction, characterized by the proportion of successful cases in each group. The criteria for successful induction of anesthesia were: (1) MOAA/S score of ≤1 after study medication administration (none or no more than two additional doses of study medication) and (2) no use of alternative anesthetic agents.  **2. *Secondary outcomes*：**The secondary outcomes of the study were: (1) time to successful induction from initial administration of the study medication, (2) time to disappearance of eyelash reflex from initial administration of the study medication, (3) BIS changes following anesthesia initiation, (4) hemodynamic changes after anesthesia initiation, and (5) use of supplementary doses of the study medication.  **3. *Safety indicators adverse events*：**Safety indicators included: (1) Injection-site pain (patients were asked about arm pain during drug injection), (2) allergic reaction, (3) bradycardia (HR ≤55 beats/min, lasting >30 seconds), (4) tachycardia (HR ≥100 beats/min, lasting >30 seconds), (5) hypotension (SBP <90 mmHg or a decrease of at least 30% from baseline), (6) hypertension (SBP ≥160 mmHg or an increase of at least 20% from baseline), (7) hypoxemia (oxygen saturation <90%, lasting >30 seconds), and (8) intubation response (unconscious swallowing, coughing, body movement, or tears).  **4. *Adverse events that require special attention*：**These included (1) incidence of blood pressure reduction requiring treatment during anesthesia, (2) incidence of respiratory depression, (3) incidence of deep or shallow anesthesia, and (4) incidence of intraoperative awareness. |
| **2、research background** |
| Recently, obesity prevalence has increased globally,^1^ which has positioned bariatric surgery, including laparoscopic sleeve gastrectomy (LSG), as a key treatment strategy.^2^ Morbid obesity leads to pathophysiological changes affecting metabolic, cardiovascular, and respiratory systems, increasing anesthetic risk.^1^ This results in prevalent perioperative complications such as circulatory depression, atelectasis, and hypoxemia.^3^ Therefore, selecting safe and effective anesthetic agents for bariatric surgery patients is vital.  Propofol is extensively utilized for anesthesia induction and maintenance, known for its rapid onset and recovery with minimal residual effects.^4^ However, it can cause dose-dependent circulatory and respiratory depression, injection pain, thereby increasing the incidence of adverse clinical drug reactions.^5^ A rare but severe complication, propofol infusion syndrome, may induce metabolic disturbances, multiple organ failure, and in extreme cases, death.^6^  Ciprofol, an innovative intravenous anesthetic developed in China, is a potent short-acting agonist of γ-aminobutyric acid (GABA) receptor with dual anesthetic and sedative functions. Compared to Propofol, it demonstrates greater potency.^7^ Preliminary studies demonstrated ciprofol’s advantageous profile, including high potency, rapid onset and recovery, absence of accumulation, and low respiratory and circulatory depression post-injection, indicating considerable clinical potential.^8,9^  However, there is limited experience with the clinical application of propofol in patients with obesity. The objective of this study was to compare the anesthetic effects and adverse reactions between ciprofol and propofol, as well as evaluate the effectiveness and safety of ciprofol for inducing general anesthesia in patients with obesity undergoing LSG.  1. Pouwels S, Buise MP, Twardowski P, Stepaniak PS, Proczko M. Obesity surgery and anesthesiology risks: a review of key concepts and related physiology. Obes Surg. 2019;29:2670–7.  2. Stenberg E, Dos Reis Falcão LF, O’Kane M, Liem R, Pournaras DJ, Salminen P, et al. Guidelines for perioperative care in bariatric surgery: enhanced recovery after surgery (ERAS) society recommendations: A 2021 update. World J Surg. 2022;46:729–51.  3. Members of the Working Party, Nightingale CE, Margarson MP, Shearer E, Redman JW, Lucas DN, et al.Peri-operative management of the obese surgical patient 2015: association of Anaesthetists of Great Britain and Ireland Society for Obesity and Bariatric Anaesthesia. Anaesthesia. 2015;70:859–76.  4. Walsh CT. Propofol: milk of amnesia. Cell. 2018;175:10–3.  5. Sneyd JR, Absalom AR, Barends CRM, Jones JB. Hypotension during propofol sedation for colonoscopy: a retrospective exploratory analysis and meta-analysis. Br J Anaesth. 2022;128:610–22.  6. Hemphill S, McMenamin L, Bellamy MC, Hopkins PM. Propofol infusion syndrome: a structured literature review and analysis of published case reports. Br J Anaesth. 2019;122:448–59.  7. Qin L, Ren L, Wan S, Liu G, Luo X, Liu Z, et al. Design, synthesis, and evaluation of novel 2,6-Disubstituted phenol derivatives as general anesthetics. J Med Chem. 2017;60:3606–17.  8. Man Y, Xiao H, Zhu T, Ji F. Study on the effectiveness and safety of ciprofol in anesthesia in gynecological day surgery: a randomized double-blind controlled study. BMC Anesthesiol. 2023;23:92.  9. Chen BZ, Yin XY, Jiang LH, Liu JH, Shi YY, Yuan BY. The efficacy and safety of ciprofol use for the induction of general anesthesia in patients undergoing gynecological surgery: a prospective randomized controlled study. BMC Anesthesiol. 2022;22:245. |
| 三、**Materials and Methods** |
| ***Study Design：***A double-blind randomized, controlled study  ***Randomization and blinding*：**The statistical team members generated 212 random numbers using SPSS 25.0 software (IBM, Chicago, IL, USA) and randomly assigned them into two groups: ciprofol (experimental group) and propofol (control group). A biostatistician, blinded to patient details, prepared sealed opaque envelopes containing each patient’s random number and group. On the day of surgery, these envelopes were opened by two researchers not involved in data collection or analysis, who then prepared the study medications based on the enclosed information. Anesthesiologists, unaware of the groups, administered the anesthesia. All data were collected by an independent researcher who was not involved in syringe preparation or data analysis. This ensured the blinding of patients, anesthesiologists, outcome investigators, and the statistician to group allocation, maintaining the study’s blinding integrity.  ***Research Center:*** The Third People’s Hospital of Chengdu  ***Sample size and Power：***We compared the efficacy of ciprofol (experimental group) with propofol (control group) in terms of the success rate for inducing general anesthesia, using a Type I error (false positive) rate of 0.025 (unilateral) and a test power of 80%. Based on a preliminary, unpublished study involving 25 patients with obesity, the success rate for inducing general anesthesia using study medications was 96%, and the non-inferiority margin was set at 8%. Using the methodology of Chow et al. [10] and R language calculations, the sample size was determined to be 95 participants per group. Considering a potential 10% loss due to follow-up issues or refusal to participate, the required sample size was increased to at least 106 individuals per group, resulting in a total sample size of 212 cases.  ***Inclusion criteria***  *Subjects who met all of the following criteria were enrolled in the trial*  1. Subjects scheduled for laparoscopic sleeve gastrectomy under general anesthesia.  2. 18 < age ≤ 65, regardless of gender.  3. Subjects with ASA score I-III.  4. Subjects with a body mass index (BMI)≥35kg/m2.  5. Subjects with blood pressure between 90-140/50-90 mmHg (inclusive); heart rate between 60-100 bpm (inclusive); body temperature between 35.4-37.5°C (inclusive); respiratory rate between 12-20 breaths per min (inclusive); SpO2 when inhaling≥92%.  6. Subjects with normal results of physical examination, laboratory tests, (routine blood/urine, blood biochemistry (including hepatic function, renal function, blood glucose, and electrolytes such as Na, K and Mg), and blood coagulation), 12-lead ECG, and abdominal ultrasonography, or abnormalities considered by the investigators to be clinically insignificant; no potential significant difficult airway problems (modified Mallampati score of I-II).  7. No previous history of primary diseases in major organs, such as the liver, kidneys, digestive tract, and blood; no history of malignant hyperthermia or other hereditary disorders; no history of mental/neurological disorders; no history of epilepsy; no contraindications for deep sedation/general anesthesia; no clinically significant history of anesthesia accidents.  8. Subjects understood the procedures and methods employed in the trial and were willing to sign informed consent forms and complete the trial in strict accordance with the designated protocol.  ***Exclusive Criteria***  *Subjects who met all of the following criteria were excluded from the trial*   1. Patients who refused to participate in the study. 2. A history of allergy or hypersensitivity to the study drugs or its excipients which would have been used in the study. 3. Subjects who had a history or evidence of any of the following diseases prior to screening/administration: 4. A history of cardiovascular disease, such as postural hypotension, serious heart valve disease, severe arrhythmia, heart failure, Adams-stokes syndrome, unstable angina pectoris; myocardial infarction within 6 months before screening, tachycardia/bradycardia requiring medications, third degree atrioventricular conductive block or a QTcF interval≥450 ms (per Fridericia’s correction formula). 5. Subjects screened for a history of bronchospasm that required treatment within the first 3 months; or subjects who developed acute respiratory tract infections, with symptoms of fever, wheezing, nasal congestion or cough within 1 week prior to baseline. 6. A history of psychiatric or neurological disorders, such as brain injury, possible intracranial hypertension, cerebral aneurysm, cerebrovascular accident history and central nervous system diseases, mental system diseases (schizophrenia, mania, insanity, etc.) and long-term history of taking psychotropic drugs. 7. *Laboratory results meeting any of the following during screening/at baseline:* 8. Abnormal liver function (ALT or AST ≥ 2.5 times the upper limit of normal value, TBIL ≥ 1.5 times the upper limit of normal value), abnormal renal function (urea ≥ 1.5 times the upper limit of normal value, serum creatinine> the upper limit of normal value, or dialysis treatment within 28 days before operation), obvious abnormal coagulation function, anemia or thrombocytopenia (HB ≤ 90g/l, PLT ≤ 80 × 109/L). 9. A positive result for any of the following markers: HBsAg, HCV-Ab, HIV-Ab, and Tp-Ab. 10. Other relevant factors: 11. Unregulated diabetes mellitus and hypertension. (Fasting blood glucose ≥11.1mmol/L during screening, and/or random blood glucose ≥ 13.6mmol/L; SBP≥160mmHg and/or DBP≥100 mmHg during the screening period). 12. Subjects who received propofol, other sedatives/anesthetics and/or opioid analgesics within 1 week prior to enrollment. 13. Had a history of drug abuse within 2 years before the screening period or had a positive result of drug screening in urine at baseline. 14. A history of alcoholism within 3 months prior to screening; alcoholism defined as an average of>2 units of alcohol per day (1 unit=360 mL beer or 45 mL liquor with 40% alcohol or 150 mL wine), or had a positive alcohol breath test result at baseline. 15. Subjects who smoked>5 cigarettes per day and had a total of>60 cigarettes within 3 months prior to screening. 16. Subjects who developed clinically significant acute disease (determined by the investigators), such as infection (respiratory tract, CNS infections, septicemia, myocarditis or endocarditis), within 2 weeks prior to screening. 17. The subjects who were judged to have difficulty in respiratory management were rated as grade IV by Modified Mallampati Score. 18. Pregnant or lactating women or subjects with birth plan within 6 months (including men). 19. Those who participated in any clinical trial as subjects within 3 months prior to screening. 20. Communication difficulties. 21. Subjects judged by the investigators to be unsuitable for participating in the trial for any reason.   **Study：**  Researchers prepared study medications and other anesthesia-inducing medications based on each patient’s lean body weight (LBW) and ideal body weight (IBW). The dosages of propofol/ciprofol, midazolam, and sufentanil were calculated using LBW for anesthesia induction. Rocuronium dosage was based on IBW. The following formulas were applied: (1 inch = 2.54 cm).  BMI (kg/m^2^) = weight (kg) ÷ height^2^ (m^2^)  LBW (kg) for males = 9720 × TBW/(6680 + 216 × BMI), where TBW is total body weight  LBW (kg) for females = 9720 × TBW/(8780 + 244 × BMI)  IBW (kg) for males = 50 + 2.3 × ([length in inches] – 60])  IBW (kg) for females = 45.5 + 2.3 × ([length in inches] – 60])  Experimental group：ciprofol  specifications：20ml:50mg  usage：They will be administered an intravenous injection of ciprofol (0.5 mg/kg) within 30 seconds.  control group：propofol  specifications: 20ml: 200mg  usage: They will be administered an intravenous injection of propofol (2.5 mg/kg) within 30 seconds.  ***Outcomes Measurements***  Trial data were collected by researchers who were blinded to the group allocation. These data included baseline characteristics, such as age, sex, height, TBW, LBW, BMI, ASA classification, prior anesthesia history, alcohol consumption habits, and comorbidities.  ***Primary outcome***  The primary endpoint was the success rate of general anesthesia induction, characterized by the proportion of successful cases in each group. The criteria for successful induction of anesthesia were: (1) MOAA/S score of ≤1 after study medication administration (none or no more than two additional doses of study medication) and (2) no use of alternative anesthetic agents.  ***Secondary outcomes***  The secondary outcomes of the study were: (1) time to successful induction from initial administration of the study medication, (2) time to disappearance of eyelash reflex from initial administration of the study medication, (3) BIS changes following anesthesia initiation, (4) hemodynamic changes after anesthesia initiation, and (5) use of supplementary doses of the study medication.  ***Safety indicators adverse events***  Safety indicators included: (1) Injection-site pain (patients were asked about arm pain during drug injection), (2) allergic reaction, (3) bradycardia (HR ≤55 beats/min, lasting >30 seconds), (4) tachycardia (HR ≥100 beats/min, lasting >30 seconds), (5) hypotension (SBP <90 mmHg or a decrease of at least 30% from baseline), (6) hypertension (SBP ≥160 mmHg or an increase of at least 20% from baseline), (7) hypoxemia (oxygen saturation <90%, lasting >30 seconds), and (8) intubation response (unconscious swallowing, coughing, body movement, or tears).  ***Adverse events that require special attention***  These included (1) incidence of blood pressure reduction requiring treatment during anesthesia, (2) incidence of respiratory depression, (3) incidence of deep or shallow anesthesia, and (4) incidence of intraoperative awareness.  ***Statistical analysis***  Primary efficacy was analyzed using the Newcombe-Wilson scoring method. [15] Differences in the success rate of inducing general anesthesia and bilateral-sided 95% confidence intervals (CI) were evaluated. If the lower limit of the 95% CI for the success rate was greater than -8%, ciprofol at a dose of 0.5 mg/kg was deemed non-inferior to propofol at a dose of 2.5 mg/kg.  The statistical analysis of other efficacy endpoints was conducted using SPSS 25.0 software. Continuous numeric variables are presented as mean ± standard deviation and inter-group comparisons were performed using the two independent samples t-test. Categorical variables are expressed as numbers and percentages (n [%]), and the chi-squared test or Fisher’s exact test was used for comparing groups. The differences were deemed statistically significant with a two-sided p-value <0.05.    ***Study procedures***  **1 Screening period (D-7 to D0 days before randomization)**: Defined D0 days on the day of study drug treatment, patients should complete the following tests and information collection before randomization of study drug.     1) Signed informed consent, collected patient demographic data;     2) Major medical history and surgical history within 5 years (or beyond 5 years but deemed necessary by the investigator), history of allergies, drug or alcohol abuse, concomitant diseases, drug combinations, birth plans, etc.     3) Physical examination: General examination (including ASA grading and modified Markov score);     4) Body weight: The total body weight (TBW) of the patients was collected (rounded to retain the whole number), and the fat-removed body weight (LBW) was calculated, with male LBW=9.27×103×TBW/(6.68×103+216×BMI). Female LBW=9.27×103×TBW/(8.78×103+244×BMI); BMI is body mass index; Anesthesia was induced by LBW.     5) Vital signs: heart rate, respiratory rate, SpO2 and blood pressure (systolic/diastolic);     6) Laboratory tests: blood routine, blood biochemistry, urine routine, pregnancy test (blood pregnancy or urine pregnancy), coagulation function;     7) 12-lead electrocardiogram examination;     8) Review patient inclusion and exclusion criteria;     9) Drug combination: The drug combination within 7 days prior to randomization should be recorded.  **2 Treatment Periods (D0)**  2.1 Preoperative Preparation (D0)  1) Reconfirm the inclusion and exclusion criteria of patients;  2) The enrolled patients were determined and randomly assigned by a random system;  3) Baseline values, including heart rate, blood pressure (systolic and diastolic), respiratory rate, SpO2 and BIS, were collected after the monitoring equipment was installed;  4) Open venous access;  5) Start mask oxygen inhalation, oxygen flow maintained at 2 ~ 4 L/min;  6) Record drug combinations/treatments;  2.2 Induction of Anesthesia (D0)    Two minutes before administering the study medications, patients were administered intravenous midazolam (0.04 mg/kg) within 15 seconds and sufentanil (0.4 µg/kg) within 30 seconds. Subsequently, they were administered an intravenous injection of either ciprofol (0.5 mg/kg) or propofol (2.5 mg/kg) within 30 seconds. If a MOAA/S score of ≤1 was not achieved within 1 minute post-initial dose, a supplementary half-dose of the initial propofol/ciprofol dose was administered over 10 seconds. A secondary supplementary dose was provided as rescue medication if a MOAA/S score of ≤1 was not achieved within 2 minutes. Failure to reach MOAA/S score of ≤1 within 3 minutes deemed the study medication unsuccessful for inducing general anesthesia, leading the anesthesiologist to induce anesthesia based on clinical judgment. Once achieving a MOAA/S score of ≤1, as indicated by the absence of response to mild stimulation or shaking, rocuronium (0.6 mg/kg) was administered intravenously within 15 seconds, followed by tracheal intubation after confirming skeletal muscle relaxation.  Baseline MOAA/S measurements were obtained prior to the administration of midazolam and re-assessed every 30 seconds thereafter. Injection pain was evaluated at 5-second intervals post-study medication administration. The time to eyelash reflex disappearance, starting from the initiation of the study medication, was measured every 5 seconds using a sterile cotton swab. During anesthesia induction, vital parameters, including ECG, HR, SpO_2_, SBP, DBP, MAP, and BIS, were closely monitored and recorded while closely observing the patients for any adverse events.  2.3 Anesthetic Maintenance (D0): The anesthesiologist determines the choice of anesthetic maintenance drugs  2.4 After the operation (D0) : Transfer to the anesthesia resuscitation room (PACU)  **技术路线**  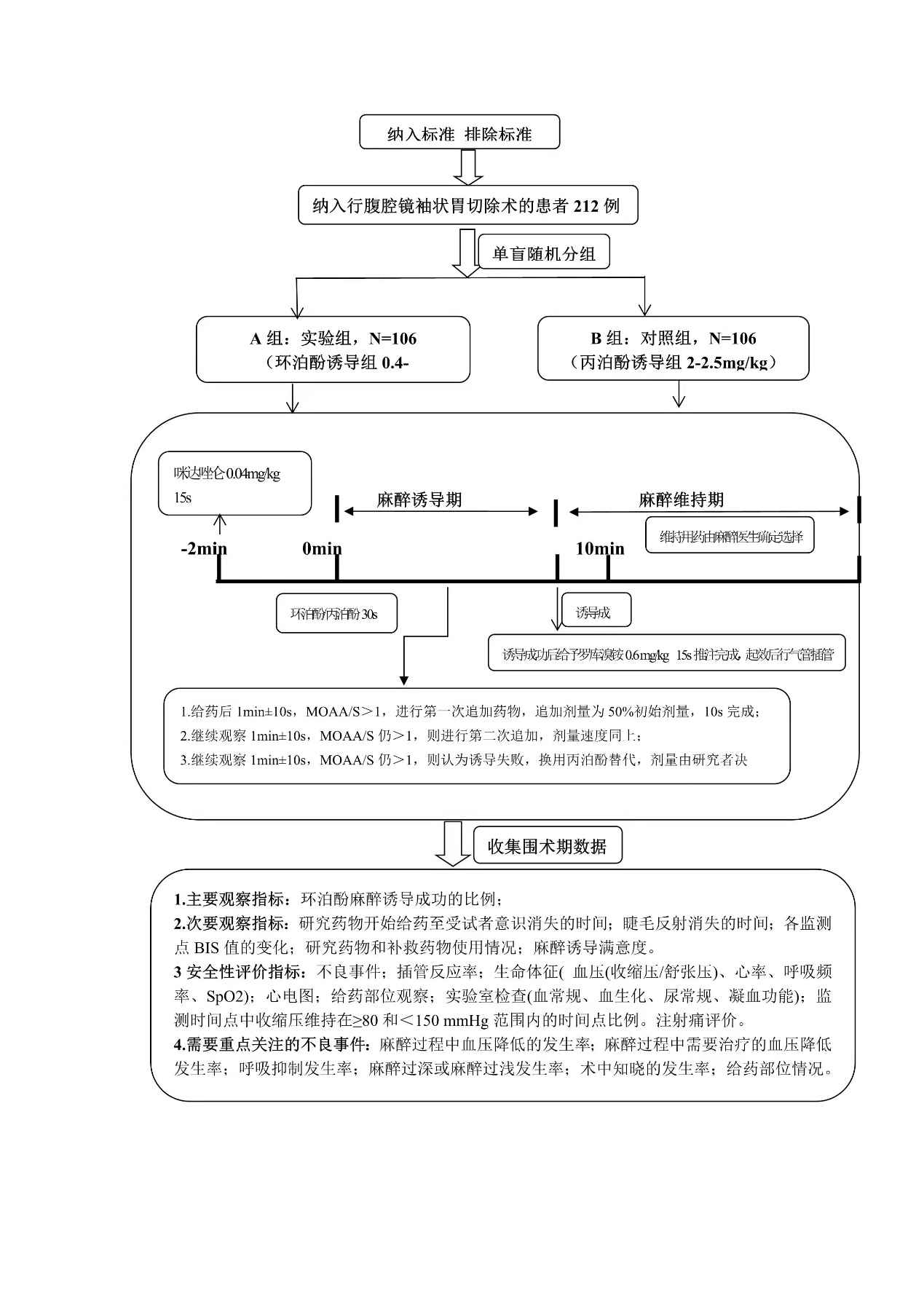  ***Plan schedule and stage objectives:***  The first stage (2022.12.01-2023.05.31): It is expected that the implementation of the clinical study will be successfully completed, and the data will be collected and analyzed  The second stage (2023.06.01-2023.12.31): Data sorting, statistics and analysis are expected to be carried out smoothly and on schedule  The third stage (2024.01.01-2024.12.01): It is expected to complete the paper writing, complete 1-2 subject papers, publish 1 SCI/Medline level article and 1 Chinese core journal article. |
| **4、Meet the construction requirements** |
| 1. Accumulated preliminary research work related to this project  The unit has carried out weight loss metabolic surgery for more than 10 years, and took the lead in establishing the Obesity and metabolic disease Center in Southwest China, which has become the center with the largest amount of metabolic weight loss surgery in Southwest China. Metabolic weight loss anesthesia team has strong strength, thus providing the research basis for this study. The weight loss anesthesia team is strong, with a sound pre-operative, intraoperative and postoperative anesthesia recording system, and a sound post-operative follow-up mechanism. All members of the research group have rich clinical experience and research experience, and can complete clinical observation and data collection related to this topic. The main researchers are reasonably equipped, have formed a research team, have a strong theoretical level and research ability, can smoothly solve the key problems in the implementation of the project, provide the necessary technical support for the implementation of the project, and can complete the relevant experimental tasks.  2. Previous research work achieved  In the early stage, we applied cipofol to painless gastroenteroscopy diagnosis and treatment, and concluded that the success rate of diagnosis and treatment of cipofol group was 100%, indicating that cipofol was comparable to propofol and reached the main endpoint. The addition times of cypofol and propofol were (0.5±0.85) and (0.7±0.71) times, respectively (P=0.052) (see Figure 1). Compared with propofol, there was no difference in the duration of successful induction of cyclopofol. As the efficacy of cyclopofol 0.4 mg/kg was stronger than that of propofol 1.5 mg/kg, the waking time and recovery time at this dose were slightly longer than that of propofol (see Figure 2). The incidence of drug-related adverse events (respiratory depression, apnea, hypoxia) in the cyclopofol group was lower than that in the propofol group, which was consistent with the results of previous studies. The duration of mesopofol apnea and hypoxia in colonoscopy was shorter than that of propofol (see Figure 3).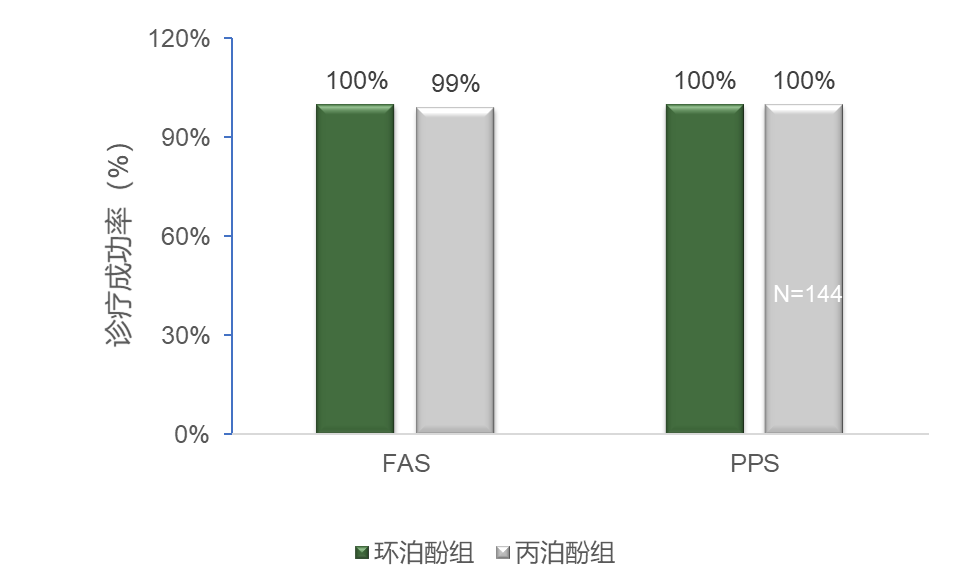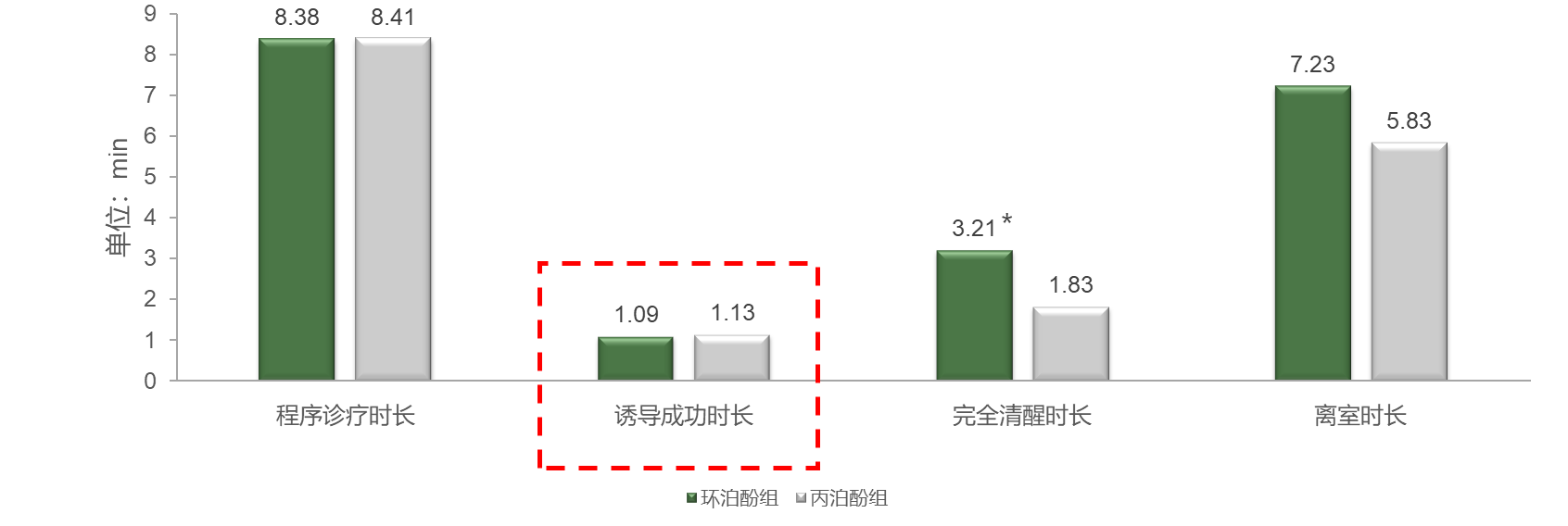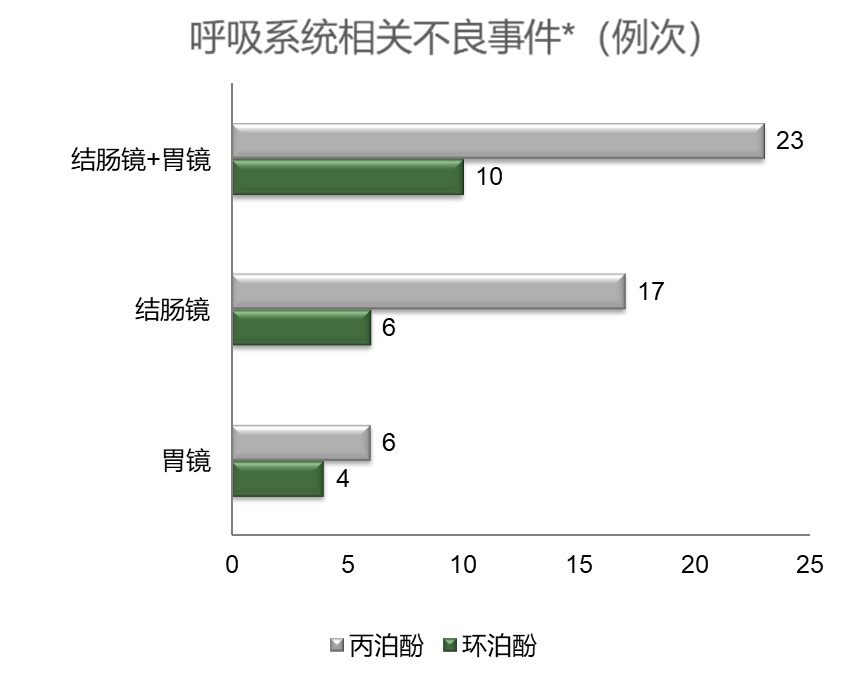 |

| **Ethical proof.** |
| --- |
|  |
| **Novelty search** |
|  |
| **review comment** |
|  |
|  |
